# The Relationship between STR-PCR Chimerism Analysis and Chronic GvHD Following Hematopoietic Stem Cell Transplantation

**Published:** 2017-01-01

**Authors:** Seyed Asadollah Mousavi, Mina Javadimoghadam, Ardeshir Ghavamzadeh, Kamran Alimoghaddam, Azadeh Sayarifard, Seyed-Hamidollah Ghaffari, Bahram Chahardouli, Ali Basi

**Affiliations:** 1 Hematology-Oncology and Stem Cell Transplantation Research Center, Tehran University of Medical Sciences, Tehran, Iran; 2 School of Medicine, Rafsanjan University of Medical Sciences, Rafsanjan, Iran; 3 Center for Academic and Health Policy, Tehran University of Medical Sciences, Tehran, Iran

**Keywords:** Hematopoietic stem cell transplantation, Chronic GvHD, Chimerism, Polymerase chain reaction

## Abstract

**Background:** The study attempts to assess the relationship between chimerism analysis using polymerase chain reaction of short tandem repeat (STR) and the incidence of chronic graft versus host disease (GvHD) as well as survival.

**Subjects and Methods:** The retrospective cohort included all patients who received allo-HSCT during 2005-2013. Data collected by day +100 were reviewed in terms of the incidence of chronic GvHD and survival. Chimerism was evaluated for whole blood, T-cell and PMN cells on days 15, 30 and 60, respectively using polymerase chain reaction of short tandem repeat (STR).

**Results:** Forty (69%) patients developed chronic GvHD, 11 (19%) relapsed and 22 (39.7%) expired during the study. There was a significant relationship between chronic GvHD and chimerism analysis including whole blood on day 60 (p=0.001), Polymorphonuclear neutrophil (PMN) on day 60 (p=0.05), T-cell on days 15 (p=0.028), 30 (p=0.01) and 60 (p=0.004). Patients with chronic GvHD showed a long-term survival as compared with those without chronic GvHD (p=0.0013).

**Conclusion:** Conducting continuous analysis of chimerism provides an opportunity to initiate immediate measures in order to prevent complications.

## Introduction

 The growing number of cancer patients has caused researchers to seek new methods of medical treatment. Hematopoietic stem cell transplantation (HSCT) is now used as a curative treatment for many benign and malignant hematologic diseases.^1^^-^^3^ Despite HSCT’s major role in treatment of cancers, its complications and problems are yet under discussion by researchers.^4^ Graft-versus-host disease (GvHD) is one of the serious complications of HSCT. Its prevalence has been reported as 20-50 percent.^5^^-^^8^

T lymphocytes of donor tissue turn to subgroups of T helper following identification of allo-antigens of patient’s cells, which cause destruction of tissue and creates symptoms of GvHD via tissue invasion and cytokine production.^9^^-^^11^ Production and release of cytokines are important in evolution and severity of GvHD.^12^^,^^13^ Chronic GvHD is a syndrome which may occur between 50 to 400 days after transplantation.^14^^,^^15^ The most important risk factor for chronic GvHD is previous acute GvHD.^16^ Using stem cell transplantation leads to increased survival in patients with malignant disease and increased number of survived patients with special medical problems such as chronic GvHD for a long time or long-term chronic GvHD.^17^ GvHD is a major barrier to successful HSCT. Even though GvHD reduces disease recurrence and increase disease free survival, it may increase likelihood of death that are unrelated to recurrence because it contributes to organ damage and vulnerability of the patient to life-threatening infections.^18^ Furthermore, successful transplantation of hematopoietic stem cells which leads to a long-term donor-derived hematopoiesis can be effective in long-term disease-free survival.^19^ Regular monitoring of patients after HSCT is crucial for transplant evaluation.^20^ One of the main goals of post-transplant follow-up is to predict negative consequences of transplantation including disease recurrence, rejection and GvHD to apply the preventive treatments, effectively. Chimerism which is known as constant presence of donor cells in the transplant recipient (host) is one of the expected consequences of transplantation.^21^ The occurrence of chimerism has been shown to be an indicator of success and durability of transplantation.^22^ Moreover, chimerism analysis can be used in the diagnosis of transplant rejection and recurrence of disease.^23^^-^^26^

The increasing number of donor cells after transplantation decrease risk of disease recurrence and its early diagnosis using chimerism analysis helps in determination of prognosis and early necessary treatments.^27^^,^^28^ The original studies on hematopoietic stem cell transplantation have shown the importance of chimerism. Now, the most useful and sensitive methods used in chimerism analysis are molecular genetics such as polymerase chain reaction based on polymorphic mini-or micro-satellite markers which can also specify very low numbers of donor and recipient cells.^29^ Since most studies have been carried out on acute GvHD.^24^^,^^30^^-^^32^ This study attempts to explore the relationship between STR-PCR-based chimerism analysis and occurrence of chronic GvHD as well as survival in a sample of Iranian patients. This study aims to be helpful in determination of interventions for patients at risk of rejection, recurrence and developing cGvHD.

## SUBJECTS AND METHODS

 Patients with allo-HSCT referred to Hematology-Oncology and Stem Cell Research Center, Tehran, Iran, in 2005-2013 were included in the retrospective study. The inclusion criteria were patients who received allo-HSCT in 2005 to 2013 and patients with complete medical records or the probability of completion by a due date. Patients with incomplete medical records or inability to complete information were excluded from the study. For collecting data, a checklist was developed based on age, sex, chimerism, acute GvHD, chronic GvHD, survival and recurrence. Patients were examined and their clinical and paraclinical data were collected during routine clinic visits following transplantation. In this retrospective study, data from allo-HSCT recipients were investigated considering the incidence of acute GvHD, chronic GvHD, recurrence and survival. In this study, chimerism was divided into two categories: complete chimerism (more than 95% of hematopoietic cells post-transplant are of donor origin) and mixed chimerism (between 5%-95% cells of donor origin in hematopoietic tissues). Patients with less than 5% donor cells developed no chimerism. After 15, 30 and 60 days of transplantation, chimerism was detected in whole blood, T-cells and PMN. The relationship between cumulative incidence of chronic GvHD and chimerism was analyzed on days 15, 30 and 60 after transplantation. Conditioning regimens employed for various types of disease were based on the HSCT protocol. GVHD prophylaxis included cyclosporine with short-term methotrexate. The limited/ extensive classification was proposed by the Seattle group.^14^

The method used for chimerism analysis in this study was polymerase chain reaction-based short-tandem repeat (STR-PCR) using 12 indicators of high differentiation. In this method three autosomal tetra nucleotide STR loci with non-overlapping allele size ranges were simultaneously amplified. Loci are, D4S2366, D16S539 and TH01. All markers used were amplified under identical PCR conditions, 200 µM of each dNTP, 20 Pmol of each oligonucleotide primers, 10 mM tris HCL (PH=8.3), 50 mM KCL, 2 mM MgCL2, 1 unit of Taq DNA polymerase (Fermantas, UK) and 100 ng of template DNA. For electrophoresis and visualization six percent polyacrylamid gels were used and DNA was visualized with DNA silver staining system.

All data were analyzed using SPSS software. T-test and the chi-square test were used for variables. Analysis of survival data was done using the software STATA, V, 11. Kaplan-Meier method, log-rank test and Cox regression model were used to determine survival. Results were considered statistically significant (p<0.05). Each patient has a corresponding code to keep all information confidential. No personal data shall is used or disclosed in any manner incompatible with the specified purposes.

## Results

 The medical records of 60 patients who received allo-HSCT in 2005-2013 were reviewed, of which two were excluded due to missing chimerism data. Finally, medical records of 58 patients were analyzed. In the collection of 58 patients, there were 37 males (63.8%) and 21 females (36.2%) with mean age of 29.3 ± 13.7 years. The distribution of patients in terms of gender, source of transplant, diagnosis of disease, the incidence of acute and chronic GvHD, recurrence and death are shown in Table 1. According to Table 1, chronic GvHD occurred in 40 patients (69%), 25 patients (62.5%) had limited GvHD and 15 (31.5%) had extensive GvHD. Among the 58 patients included in the study, 11 (19%) relapsed and 22 (39.7%) expired.

Table 2 shows the number and percentage of chronic GvHD patients who developed skin, mucosal, liver and gastrointestinal tract involvement. As indicated, skin and mucosa are the most affected sites (87.5%).

Among patients with chronic GvHD, 35 (67%) patients received peripheral blood and 5 (83%) received bone marrow. There was no significant difference between these two groups (p=0.67).

Table 3 shows relationship between chronic GvHD and chimerism. There is a significant relationship between chronic GvHD and chimerism analysis including whole blood on day 60 (p=0.001), PMN on day 60 (p=0.05) and T-cell on days 15 (p=0.028), 30 (p=0.01) and 60 (p=0.004)*.* Patients with complete or full donor chimerism may be at increased risk of developing chronic GvHD compared to those with low donor cell chimerism.

**Table 1:** Distribution of patients in terms of gender, source of transplant, diagnosis of disease, the incidence of acute and chronic GvHD, recurrence and death

**Variable**		**Number (%)**
**Gender**	Female	21 (36)
	Male	37 (64)
**Diagnosis**	CML	6 (10.3)
	Thalassemia	17 (29.3)
	ALL	10 (17.2)
	AML	14 (24.2)
	Aplastic anemia	5 (8.5)
	other	6 (10.2)
**Sources of transplantation**	PB	52 (89.7)
	BM	6 (10.3)
**Acute GvHD**	Yes	36 (62.1)
	No	22 (37.9)
**Chronic GvHD**	Yes	40 (69)
	No	18 (31)
**Chronic GvHD type**	Limited	25 (62.5)
	Extensive	15 (37.5)
**Recurrence**	Yes	11 (19)
	No	47 (81)
**Death**	Yes	23 (39.7)
	No	35 (60.3)

**CML:** Chronic Myelogenous Leukemia, **ALL:** Acute Lymphoblastic Leukemia, **AML:** Acute Myeloid leukemia, **PB:** Peripheral Blood, **BM:** Bone Marrow, **GvHD:** Graft-versus-Host Disease

**Table 2:** The number and percentage of chronic GvHD patients who developed skin, mucosa and liver and gastrointestinal tract involvement

**Involved organ**		**Number (%)**
**Skin and Mucosa**	Yes	35 (87.5)
	No	5 (12.5)
**Liver**	Yes	9 (77.5)
	No	31 (22.5)
**Gastrointestinal**	Yes	1 (2.5)
	No	39 (97.5)

**Figure 1:**
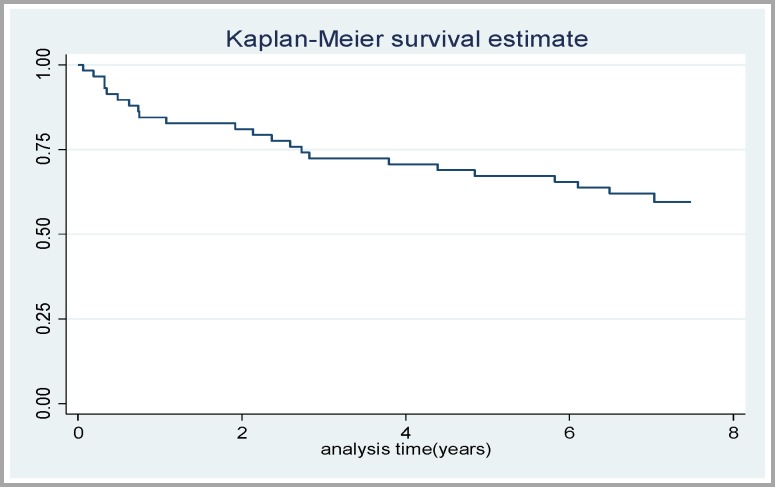
Patients’ survival

**Figure 2:**
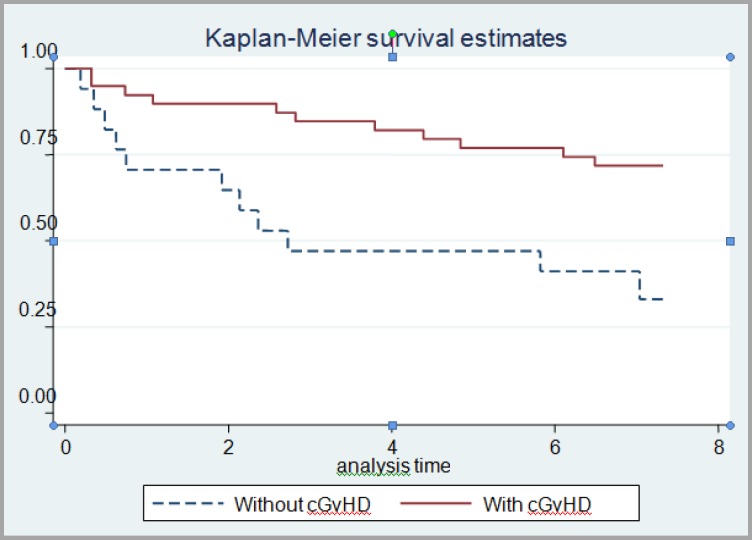
Patients’ survival in term of chronic GvHD incidence

Patients were followed-up for a mean period of 85 months. The overall survival has been shown in Figure 1. The mean survival time cannot be reported as the curve has not been reached to 50%. One, three and five-year survival in patients were 0.85%, 0.70% and 0.65%, respectively (Figure 1). Although men had higher survival than women, the difference was not significant (p=0.261, log rank test). Survival rate (Figure 2) in patients with chronic GvHD was significantly higher than patients without chronic GvHD (p=0.0013).

**Table 3:** The relationship between chronic GvHD and chimerism in terms of blood cell type and the day of chimerism analysis

			**Chimerism State**			**p-value**
** Day**		**cGvHD**	**Complete**	**Mix**	**Without chimerism**	
**Whole ** **blood ** **chimerism**	15	Yes	29 (70%)	10 (72%)	1 (50%)	0.88
		No	13 (30%)	4 (28%)	1 (50%)	
		Total	42 (100%)	14 (100%)	2 (100%)	
	30	Yes	28 (72%)	12 (67%)	0 (0%)	0.69
		No	11 (28%)	6 (33%)	0 (0%)	
		Total	39 (100%)	18 (100%)	0 (0%)	
	60	Yes	37 (80%)	3 (30%)	0 (0%)	0.001*
		No	9 (20%)	7 (70%)	0 (0%)	
		Total	46 (100%)	10 (100%)	0 (0%)	
**T cell ** **chimerism**	15	Yes	26 (84%)	13 (52%)	1 (100%)	0.028*
		No	5 (16%)	12 (48%)	0 (0%)	
		Total	31 (100%)	25 (100%)	1 (100%)	
	30	Yes	28 (80%)	12 (55%)	0 (0%)	0.01*
		No	7 (20%)	10 (45%)	1 (100%)	
		Total	35 (100%)	22 (100%)	1 (100%)	
	60	Yes	33 (82%)	7 (44%)	0 (0%)	0.004*
		No	7 (18%)	9 (56%)	0 (0%)	
		Total	40 (100%)	16 (100%)	0 (0%)	
**PMN cell ** **chimerism**	15	Yes	32 (71%)	7 (64%)	1 (100%)	0.8
		No	13 (29%)	4 (36%)	0 (0%)	
		Total	45 (100%)	11 (100%)	1 (100%)	
	30	Yes	38 (73%)	2 (40%)	0 (0%)	0.07
		No	14 (27%)	3 (60%)	1 (100%)	
		Total	52 (100%)	5 (100%)	1 (100%)	
	60	Yes	38 (76%)	2 (33%)	0 (0%)	0.05*
		No	12 (24%)	4 (67%)	0 (0%)	
		Total	50 (100%)	6 (100%)	0 (0%)	

**PMN:** Polymorphonuclear neutrophil

* **: **statistically significant at p<0.05

## Discussion

 In present study, acute GvHD occurred in 62.1% and chronic GvHD in 69%. Limited chronic GvHD occurred with the highest incidence among the patients. The most commonly involved organs with chronic GvHD are skin, mucous membrane and liver. Relapses occurred in 20% of patients and 40% expired during the study.

**cGvHD:** Chronic Graft-versus-Host Disease

In this study, a significantly higher incidence of chronic GvHD was seen in patients with complete chimerism (whole blood on day 60, PMN on day 60 and T cell on days 15, 30 and 60). The results of this study are consistent with the findings of a study by Barrios and Rupa-Matysek that found the kinetic assessment or long-term chimerism to be more important than absolute value of single measurement of chimerism. Moreover, regular monitoring of chimerism by the subgroups of T cell CD3^+^ is an efficient method in predicting the risk of developing GvHD.^33^^,^^34^

A study conducted by Mossallam indicated that low T cell chimerism was significantly associated with a reduced risk of chronic GvHD.^35^

The results of this study are similar to the findings obtained by Sairafi and pasquet, which indicated that the incidence of chronic GvHD is higher in patients with complete chimerism.^22^^-^^24^

These results indicated that there was a direct association between the incidence of chronic GvHD and survival of patients (p=0.0013). Research conducted by Prez et al. showed that survival of patients who developed chronic GvHD after nonmyeloablative allogeneic transplantation was higher than those without chronic GvHD within 24 months.^36^ Barrios showed that in patients with leukemia death rate was significantly lower than those who developed chronic GvHD after stem cell transplantation.^33^

One advantage of this study was the ability of the retrospective cohort design to measure exposure and outcome in context of time. Another aspect was the checklist developed based on individual patient data. This study has considerable limitation, including small sample size and lack of specific target population. Due to the fact that patients were from one referral center, results of this survey may not be generalizable to larger population of patients.

## CONCLUSION

 Conducting continuous analysis of chimerism after allo-HSCT is useful in predicting the incidence of chronic GvHD, prognosis and survival. Chimerism analysis also provides an opportunity to initiate immediate measures to prevent complications.

Due to lack of similar studies and differences in blood components in various studies, further research is recommended to focus on specific blood cells to predict the risk of cGvHD recurrence and death in patients receiving allo-HSCT.
